# Effect of Transcutaneous Tibial Nerve Stimulation in Multiparous Egyptian Women with Vaginal Laxity: A Randomized Controlled Trial

**DOI:** 10.1007/s10508-025-03093-7

**Published:** 2025-02-10

**Authors:** Doaa A. Abdel Hady

**Affiliations:** Department of Physical Therapy for Women’s Health, Deraya University, Minia, 61511 Egypt

**Keywords:** Transcutaneous tibial nerve stimulation, Pelvic floor exercises, Vaginal laxity, Sexual dysfunction, Multiparous

## Abstract

Vaginal laxity (VL) is a common symptom of pelvic floor disorder, an underreported condition, and has a substantial impact on women's sex lives and relationships. The study aimed to determine the effect of transcutaneous tibial nerve stimulation (TTNS) on women with VL. Fifty women with vaginal laxity were chosen from an outpatient clinic in Egypt. They were randomly split into two equal categories (A and B). Group A (*n* = 25) received pelvic floor exercises (PFE), while Group B (*n* = 25) received TTNS and PFE. Every week, both groups performed three sessions for three months. The outcomes were evaluated both before and following treatment using ultrasound imaging to assess pelvic floor muscle (PFM) function and the Female Sexual Function Index to measure sexual function. Vaginal Laxity Questionnaire (VLQ) were used to examine vaginal looseness. The analysis demonstrated a significant improvement in vaginal laxity in both groups. Post-treatment comparisons revealed a statistically significant difference in VLQ scores and PFM strength between Groups B and A. These findings indicate that TTNS is notably more effective in improving PFM activity and VLQ scores in women with vaginal laxity.

## Introduction

Vaginal laxity (VL) is defined as vaginal flaccidity and lack of elasticity (Haylen et al., 2010). VL remains a neglected disorder among urogynecologists, with reports of discomfort and satisfaction as a result of decreased sexual sensation, which can affect sexual function and relationships (Pauls et al., 2012). Pelvic floor muscles (PFM) can grow weaker or damaged, and the vaginal walls might stretch or enlarge, resulting in decreased satisfaction during sexual activity, difficulty achieving orgasm, and a lack of confidence (Kamisan Atan et al., 2015; Toozs-Hobson et al., 2008). It is observed in 24% to 38% of women. It is caused by vaginal birth, pregnancy, delivery, menopause, obesity, and pelvic organ prolapse (Newman et al., 2018; Pauls et al., 2012). During pregnancy and vaginal birth, trauma to the pelvic floor and vagina may result in an increased diameter of the vaginal orifice, causing changes in sexual sensitivity, behavior, and physiological responses during sexual activity (Sekiguchi et al., 2013).

Non-surgical approaches to managing VL include behavioral therapy, hormone replacement therapy, and the use of medical treatments such as firming creams (Karcher & Sadick, 2016). Surgical options are also available to reduce VL; however, they carry significant risks, including tissue scarring, nerve damage, and potential adverse effects on sensation. Additionally, surgical management is often associated with dyspareunia and extended recovery periods (Dobbeleir et al., 2011). Many experiments have been conducted to search for a noninvasive, safe, and effective treatment with fewer complications as it has been associated with surgical procedures (Lee, 2014). Additionally, a survey of obstetricians and gynecologists revealed that 83% of patients needing surgical treatment were worried about the risk for postoperative dyspareunia (Toozs-Hobson et al., 2008).

PFM plays an important role in maintaining vaginal tone. These changes have a substantial impact on women's quality of life and are beneficial to their sexual lives (Sekiguchi et al., 2013). So, PFM training may have an effect on hypertensive muscles in women with VL (Zielinski et al., 2012). Pelvic floor exercises (PFE) and levator ani muscle contraction appear to have a major impact on their lives and boost sexual responsiveness. Pelvic floor therapy and muscle strengthening in that area have a major impact on their sexual activity. PFE may help increase muscle tone in people who suffer from VL (Bø et al., 2000).

Having a vaginal tone is also part of the role of PFM. This is an essential change that has a strong influence on the quality of life of women and is helpful for sexual life (Sekiguchi et al., 2013). Thus, in women with VL (Zielinski et al., 2012), Proper PFM training especially contraction of the pelvic floor and levator ani muscle seems to influence their lives greatly giving them positive sexual responsiveness. What affects their sexual activity the most is pelvic floor therapy and strengthening those muscles. In patients with VL, PFE may contribute to increased muscle tone (Bø et al., 2000).

Neuromodulation (NM) is the change of nerve activity through feedback from electrodes placed near the nerve. It is intended to treat the functions and clinical symptoms such as pain relief, return of bowel movement, and bladder control (Lemos & Lemos, 2016). Impulses are generated by NM stimulation via transcutaneous pelvic (Lauretti et al., 2015; Wang et al., 2009), transvaginal and transanal stimulation (Bendaña et al., 2009), transcutaneous tibial nerve stimulation (TTNS) (Istek et al., 2014; Tirlapur et al., 2013), sympathetic nervous system (Everaert et al., 2001; Fariello & Whitmore, 2010; Martellucci et al., 2012), pudendal nerve stimulation (Carmel et al., 2010; Peters et al., 2015), or conus medullary stimulation (Buffenoir et al., 2015). Non-pharmacological management of PFM dysfunction: NM is effective in alleviating pelvic pain and may help improve various gastrointestinal, urinary, or sexual functions that can be associated with VL (Kelly et al., 2016). NM increases PFM tone by increasing the number of vesicles of neurotransmitter degranulated at the neuromuscular interface with each wave potential, leading to enhanced muscle contraction (Logsdon et al., 2006; Malaguti, 2009; Qian & Delaney, 1997).

TTNS is a non-ablative, reversible, and minimally invasive therapeutic that consists of electrically or chemically inhibiting, stimulating, modifying, regulating, or therapeutically altering activity in the central nervous system, peripheral nervous system, or autonomic nervous system (Krames et al., 2009). As far as we know, clinical studies have never been conducted to evaluate the positive effect of TTNS in VL. Nevertheless, trials have shown excellent tolerability as measured by reproducible improvements in vaginal tightness and the use of PFM training for sexual function and sexual pain (Pereira et al., 2021). We specifically expected that TTNS on multiparous women with VL would be helpful in these conditions.

## Method

The research was planned as a randomized, controlled trial (single-blinded, two parallel arm groups). It lasted from January 1 to June 30, 2023. Based on force data derived from a pilot study conducted on 5 subjects in each group, the sample size was calculated using G*POWER software for statistical analysis (version 3.1.9.2; Franz Faul, Universitat Kiel, Germany), and it was determined that the sample size that was needed for this research was 25 subjects in each group. The calculation was 0.05, power = 80%, and effect size = 0.81.

### Participants

A total of 50 women were selected to participate in the research. Women with VL were recruited through commercials and referred to the trial through the gynecology outpatient clinic at Deraya University, based on gynecologist diagnoses and recommendations.

Females with VL were chosen at random from a clinic for outpatients. The age of the participants ranged from 30 to 40 years; their BMI was 25–30 kg/m^2^, and they had three normal vaginal deliveries. They were randomly separated into two groups (A and B). PFM exercises were given to Group A, and TTNS and PFE were delivered to Group B.

Inclusion criteria were participants had a history of vaginal winds, lack of sensation during sexual activity, lack of friction during sexual activities, and labia laxity. The Vaginal Laxity Questionnaire (VLQ) was administered to women who agreed to participate. Exclusion criteria were females with previous experience of vesicovaginal fistula, untreated uterine bleeding, infection of the urinary tract, pre-diabetic or diabetes, intrauterine device, chest cavity and/or heart disease, and any type of tumor; cervical dysplasia; previous active vaginal infection; and metabolic syndrome with compensatory responses; patients undergoing pelvic floor rehabilitation; patients with a cognitive deficiency; and peripheral or systemic neurological disease.

All women received notice of the nature, objectives, and advantages of the study, as well as the freedom to opt out or discontinue the study at any time and to maintain the privacy of any data collected. The randomization process is carried out by a computer system in a one-to-one allocation ratio (block size 4). The trial numbers (1. TTNS and PFE and 2. PFME) were placed in opaque protective envelopes that study individuals opened following the signing of the consent form. The researchers who helped with questionnaire completion, as well as the investigators in charge of performing ultrasonography and data analysis, were blinded to the therapy group to which the participants were randomly assigned. They were receiving an initial evaluation before the first session. Females were split into two groups (A and B). The blinding continued throughout the study, with the investigators responsible for data processing and patients unaware of the group assignment. The statisticians and outcome assessors were provided no information about the patients.

**Fig. 1:**
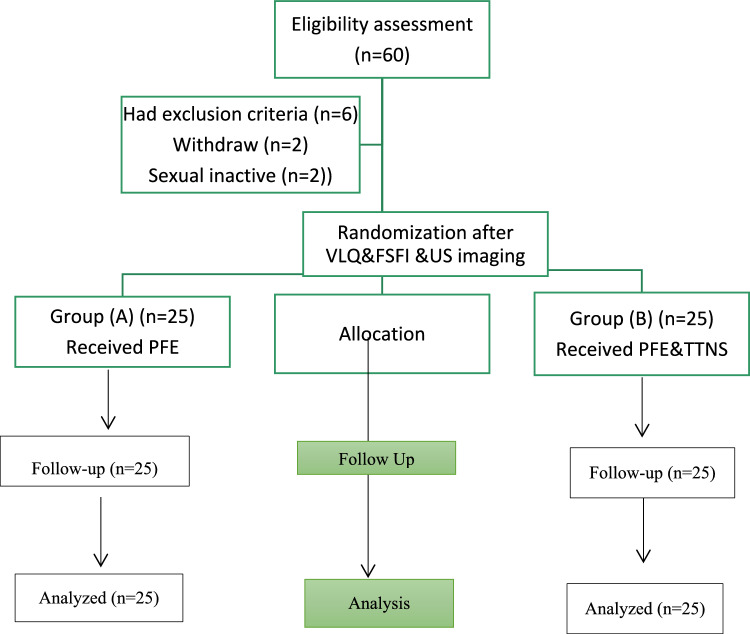
Study chart

Participants were followed up six and twelve weeks after the interventions. Figure 1 indicates the flow chart of the study.

### Procedure and Measures

*Pelvic floor exercises (PFE)*: Groups A and B were given pelvic floor exercises (PFE). Each patient was shown how to carry out PFE correctly and rest in crock lying. At the start of the PFE, we were to determine the participant's PFM and conduct the correct muscle contraction using vaginal palpation and teaching materials. This was followed by rapid, gradual, and slow contractions of PFM for strengthening and rehabilitation. Then, PFE was progressed every three weeks of intervention, with three progressions (6 repetitions maintained for 6 s, 6 s of rest, 8 repetitions sustained for 8 s, 8 s of a break, and 10 repetitions sustained for 10 s, followed by a 10-s rest). All participants were instructed to relax their abdominal and gluteal muscles, not strain, and to avoid holding their breath during PFM contractions. Participants were instructed not to carry out any PFE other than those described in the current study's intervention protocol (Cavkaytar et al., 2015).

*Transcutaneous tibial nerve stimulation (TTNS)*: Group (B) received an electrical stimulation device with a balanced, asymmetric pulsed current with a constant voltage, type 5200, for 30 min, while the patient remained supine. The electrodes were attached to the ankle, one just beyond the medial malleolus and one approximately 10 cm above. To ensure that the electrodes engaged the tibial nerve, a 10 Hz frequency and a pulse length of 200 s were first used, followed by a gradual rise in intensity to demonstrate a rhythmic hallux bending movement of the big toe (Padilha et al., 2020).

Before and three months after the intervention, the two groups (A and B) were evaluated.

An ultrasonic imaging device (Mindray DP10, B-mode, serial number: bn-75013216, from China) with a frequency of 5 MHz was used to assess the mobility (strength) of voluntary PFM contractions in every patient. It has strong inter-rater reliability (ICC, 0.81, 0.7123) and intra-rater reliability (ICC, 0.98, 0.9841) for assessing PFM mobility (Mørkved et al., 2004). The findings were obtained with a full bladder. With the woman in crock lying at an angle of roughly 60° from vertical, the ultrasonic transducer was placed transversely across the midline of the abdomen, directly above the symphysis pubis (Arab et al., 2010).

Women completed three maximal PFM contractions to evaluate the movement of the posterior bladder border caused by a PFM contraction. For the measurement, a well-defined border is at the moment of greatest visible displacement throughout the movement. The shot was taken at the highest point of displacement. The woman relaxed the PFM at this point. To guarantee that the angle remained constant between the rest and the curve peak, the probe was not changed during evaluation (Arranz-Martín et al., 2021).

#### Vaginal Laxity Questionnaire

Determine the rating range of 1 to 7, with 1 indicating “very loose,” 2 indicating “moderately loose,” 3 indicating “slightly loose,” 4 indicating “neither loose nor tight,” 5 indicating “slightly tight,” 6 indicating “moderately tight,” and 7 indicating “very tight.” VLQ is not a validated tool, although no validated patient-diagnostic tool for VL exists at present (Krychman et al., 2017).

#### Arabic Female Sexual Function Index (FSFI)

The Arabic FSFI is an accessible and thorough 19-item self-reporting instrument. It was used to measure the six main elements of female sexual function in both groups: desire, arousal, lubrication, orgasm, patient satisfaction, and discomfort. The questionnaire had many response options: the initial two inquiries had five possible responses, while the following questions were rated on a scale of zero to five. To establish the scores, the replies were aggregated and multiplied by certain factors (0.6 for questions 1 and 2, 0.3 for questions 3–10, and 0.4 for questions 11–19). The grading scale ranged from 2 to 36, and a result of less than 28.1 indicated an impairment in sexual activity (Anis et al., 2011).

The reliability, validity, and local acceptance of the Arabic FSFI as a measure for measuring female sexual dysfunction in the population of Egypt have been demonstrated. The total score and each domain score have test–retest reliability ranging from 0.92 to 0.98. Internal consistency was strong across domains, with scores ranging from 0.85 to 0.94. The Arabic FSFI also revealed good discriminant validity. Overall, the Arabic FSFI performed well, with an AUC of 0.98 and a 95% confidence interval (CI) ranging from 0.97 to 0.99 (Anis et al., 2011). The therapy used in all groups consisted of three sessions per week for three months.

### Statistical Analyses

The data analysis was carried out by a statistician who was unaware of the group allocation and had no participation in the study. The age and BMI of the groups were compared using an unpaired t test. The Shapiro–Wilk test was used to ensure that the data were distributed normally. To assess group homogeneity, Levene's test for variance homogeneity was used. A mixed MANOVA was used to compare the effects of force and FSFI within and between groups. For future multiple comparisons, post hoc tests using the Bonferroni correction were used. The Mann–Whitney test was used to compare VLQ between groups, and the Wilcoxon signed-rank test was used to compare pre- and post-therapy. The level of significance was fixed at *p* < .05 for all statistical tests. SPSS software version 25 for Windows (IBM SPSS, Chicago, IL, USA) was used for all statistical analysis.

## Results

### Participant Characteristics

Table 1 shows the participant features of Groups A and B. There was no significant difference in age or BMI between the two groups (*p* >.05).

**Table 1:** Participant characteristics

	Group A	Group B	MD	*t*-value	*p*-value
	Mean ± SD	Mean ± SD			
Age	37.48 ± 3.44	35.04 ± 5.33	− 0.56	− 0.40	.68
BMI	27.47 ± 2.03	27.41 ± 1.61	0.06	0.13	.89

*SD* standard deviation, *MD* mean difference,* p*-value, probability value

### Intervention Effect on Pelvic Floor Muscle Strength and Female Sexual Function Index After 12 Weeks

The intervention had a significant interaction with time on PFM strength and FSFI (*F* = 4.16, *p* = .02, = 0.15). There was a significant main impact of time (*F* = 130.52, *p* = .001, = .84). There was a substantial treatment effect (*F* = 8.18, *p* = .001, = .26).

PFM strength and FSFI increased significantly in Groups A and B after therapy compared to pre-treatment (*p* > .001). The percent change in PFM and FSFI for Group A was 45.45 and 31.20%, respectively, whereas for Group B it was 58.33 and 37.95% (Table 2).

**Table 2:** Mean PFM strength and FSFI before and after intervention in Groups A and B after 12 weeks

	Group A	Group B	MD (95% CI)	*p* value
	Mean ± SD	Mean ± SD		
PFM strength (cm)				
Before treatment	0.11 ± 0.04	0.12 ± 0.03	− 0.01 (− 0.02: 0.01)	.69
After treatment	0.16 ± 0.03	0.19 ± 0.04	− 0.03 (− 0.05: − 0.01)	.002
MD (95% CI)	− 0.05 (− 0.06: − 0.03)	− 0.07 (− 0.09: − 0.06)		
% of change	45.45	58.33		
	*p* = .001	*p* = .001		
FSFI				
Pre-treatment	19.33 ± 2.37	20.13 ± 2.50	− 0.8 (− 2.19: 0.58)	.25
Post-treatment	25.36 ± 1.67	27.77 ± 2.17	− 2.41 (− 3.51: − 1.31)	.001
MD (95% CI)	− 6.03 (− 7.35: − 4.71)	− 7.64 (− 8.95: − 6.32)		
% of change	31.20	37.95		
	*p* = .001	*p* = .001		

*SD*, Standard deviation, *MD* Mean difference, *CI* Confidence interval;* p*-value, probability value

### Between-Group Comparison

There was no significant difference between the groups before therapy (*p* > .05). After therapy, Group B had significantly higher PFM strength and FSFI than Group A (*p* < .01) (Table 2).

### Effect of Treatment on Vaginal Laxity Questionnaire After 12 Weeks

VLQ in Groups A and B increased significantly after therapy compared to before treatment (*p* > .001). The VLQ of Group B increased significantly more than that of Group A (*p* .001) (Table 3).

**Table 3:** Median values of VLQ pre- and post-treatment of Groups A and B

VLQ	Group A	Group B	*U*-value	*p* value
	Median (IQR)	Median (IQR)		
Pre-treatment	2 (2–1)	2 (2–1)	286.50	.58
Post-treatment	3 (4–2)	5 (5–4)	107	.001
Z-value	− 3.74	− 4.42		
	*p* = .001	*p* = .001		

*IQR* interquartile range;* U*-value, Mann–Whitney test value;* Z*-value, Wilcoxon signed ranks test value* p*-value, probability value

## Discussion

The purpose of this study was to examine TTNS as a non-surgical therapy option for women with VL. In those with sexual dysfunction, there is a connection between multifidus (MF), diaphragm, and PFM strength (Abdel Hady & Abd El-Hafeez, 2024; Hady et al., 2024b). Research has shown that PFM dysfunction increases female sexual satisfaction, implying a link between PFM performance and female sex functions. One of the pelvic floor disorders is VL, which is still seldom addressed by patients and their healthcare providers, and the absence of evidence-based therapy hurts the condition's management.

The current study found that there was a significant increase in force and FSFI in Groups A and B after the intervention compared to before. Group A's percent change in force and FSFI was 45.45 and 31.20%, respectively, while Group B's was 58.33 and 37.95%. In addition, the researchers found a significant increase in VLQ in Groups A and B after therapy compared to pre-treatment. The VLQ of Group B increased significantly more than that of Group A.

Citak et al. (2010) showed that PFE had a significant increase in every factor of the FSFI in postpartum Turkish women. PFE reduces urine leakage, enhances female sexual life and function, and boosts PFM strength by facilitating PFE. Hwang et al. (2020) found that exercises are done to strengthen the months, therefore describing methods toward some factors related to the above PFM, including per-women capability to achieve utmost orgasm maximal and also the degree of orgasm; multiple orgasms; and vaginal sensation during intercourse. Hence, PFM strengthening activities confirm the enhanced sexual self-confidence of women as an effect in this study (Golmakani et al., 2015). Kegel training leads to strength of the levator ani muscle due to providing support and reducing the burden on the ligament while elevating muscular activity. And, these exercises help in improving the blood flow to the area (Golmakani et al., 2015). However, this was supported in the study conducted by Kershaw et al., (2019), and in females found that PTNS increased desire, orgasm, libido, satisfaction, and discomfort in the pelvis. TTNS is a safe and effective treatment for women with sexual dysfunctions. FSFI was reported to be significantly higher in the subcategories of lubrication, arousal, and orgasm (Lúcio et al., 2014; Zimmerman et al., 2018).

TTNS causes changes in the neuromechanical arrangement of neurons. It is also believed that peripheral nerves representing the same sacral region (S2 and S3) as the pudendal nerve may activate the PFM. As a result, it stimulates and modulates the neural circuit's function (Padilha et al., 2020). Also, parasacral transcutaneous electrical stimulation and the Knack Technique help to reduce VL while also enhancing PFM and sexual function (Hady et al., 2024a). This study will help to provide a better understanding of therapy options for this illness, which has had a negative influence on women's sexual activities. This research has various strengths since it targets an important issue with a high prevalence among women. Additionally, the study assessed the outcomes using objective methods. There have been multiple reports of women with VL experiencing discomfort, bleeding, and burning sensations for up to 5 days after laser treatment. Also, the study method is inexpensive, simple to administer, has few adverse effects, and stimulates sexual activity.

The study had certain limitations, including the absence of a follow-up period to assess the long-term effects of TTNS used after 3 months, and conducting subsequent trials for a longer duration, which could potentially limit generalizability because not all subjects did not raise functional level to sexual function. Nevertheless, it seems that sexual function is improved although, in Group B, nine women reached the normal and, in Group A, four women reached the normal. But the VLQ got back to normal pretty quickly already. A validated patient-diagnostic tool for VL does not currently exist, either.

In conclusion, this study determined that TTNS is more effective in alleviating VL symptoms and enhancing pelvic floor performance. As a result, these findings could contribute to refining treatment guidelines for PFM underactive, VL, and sexual disorders.

## Data Availability

Not available.
